# Effectiveness and Safety of Chemotherapy Combined with Dendritic Cells Co-Cultured with Cytokine-Induced Killer Cells in the Treatment of Advanced Non-Small-Cell Lung Cancer: A Systematic Review and Meta-Analysis

**DOI:** 10.1371/journal.pone.0108958

**Published:** 2014-09-30

**Authors:** Rui-xian Han, Xu Liu, Pan Pan, Ying-jie Jia, Jian-chun Yu

**Affiliations:** 1 First Teaching Hospital of Tianjin University of Traditional Chinese Medicine, Tianjin, China; 2 Tianjin University of Traditional Chinese Medicine, Tianjin, China; Shanghai Jiao Tong University School of Medicine, China

## Abstract

**Background:**

Lung cancer, particularly non-small-cell lung cancer (NSCLC) is the leading cause of cancer mortality. Chemotherapy combined dendritic cells co-cultured with cytokine-induced killer cells (DC-CIK) immunotherapy has been applied in advanced NSCLC patients' treatment, but couldn't provide consistent beneficial results. Therefore, it is necessary to evaluate the efficiency and safety of combination therapy to promote the application.

**Methods:**

A literature search for randomized controlled trials of NSCLC was conducted in PubMed database. Before meta-analysis was performed, studies were evaluated heterogeneity. Pooled risk ratios (RRs) were estimated and 95% confidence intervals (CIs) were calculated using a fixed-effect model. Sensitivity analysis was also performed.

**Results:**

Six eligible trials were enrolled. Efficiency and safety of chemotherapy followed by DC-CIK immunotherapy (experimental group) and chemotherapy alone (control group) were compared. 1-year overall survival (OS) (*P* = 0.02) and progression free survival (PFS) (*P* = 0.005) in the experimental group were significantly increased compared with the control. Disease control rate (DCR) (*P* = 0.006) rose significantly in experimental group. However, no significant differences between the two groups were observed in 2-year OS (*P* = 0.21), 2-year PFS (*P* = 0.10), overall response rate (ORR) (*P* = 0.76) and partial response (PR) (*P* = 0.22). Temporary fever, anemia, leukopenia and nausea were the four major adverse events (AEs) treated by chemotherapy. The incidence of anemia, leukopenia and nausea in the experimental group was obviously lower than the control group. Temporary fever rate was higher in experimental group than that in the control, but could be alleviated by taking sufficient rest.

**Conclusions:**

Chemotherapy combined with DC-CIK immunotherapy showed superiority in DCR, 1-year OS and PFS, and no more AEs appeared, however, there was no significant improvement in ORR, PR, 2-year OS and PFS. As a whole, the combination therapy is safer but modest in efficacy for advanced NSCLC patients.

## Introduction

Lung cancer has been considered as one of the most commonly diagnosed type of cancer affected by population aging and growth as well as change in lifestyle, such as smoking and physical inactivity [1]. Furthermore, lung cancer is a devastating disease, particularly non-small-cell lung cancer (NSCLC); NSCLC is among the leading causes of mortality worldwide and accounts for approximately 80% to 85% of all lung cancer cases [2].

Surgery, radiation and chemotherapy are the three most widely employed cancer treatments; however, these treatments elicit multiple side effects and often fail to completely remove the tumor tissues, including small lesions and metastatic cells that may cause disease recurrence after treatment [3]. In chemotherapy, platinum-based regimens are considered as the most important form of treatment [4]. For example, a four-cycle regimen (i.e., cisplatin or carboplatin) is administrated, thereby improving the conditions of patients with NSCLC. However, five-year survival rate remains very poor, drug resistance and adverse effects appears subsequently, thus, the more effective and safer treatments are urgently require to prompt to improve the quality and duration of life. With progression in disease treatments, immunotherapy, particularly dendritic cells co-cultured with cytokine-induced killer cell (DC-CIK) therapy, has been applied as an important component of cancer treatment [5].

*Ex vivo* and *in vivo* experimental evidence has shown that CIK cells [6], which are cytotoxic lymphocytes generated from peripheral lymphocytes by a cytokine cocktail containing anti-CD3 monoclonal antibody, IFN-γand IL-2 and mainly consist of the CD3^+^CD56^+^ subset [7], can be used against solid tumors. These cells show a high level of cytotoxic activity and lyse a broad range of tumor cell lines, including multi-drug resistant and autologous tumor cells [8].

These biological features of CIK cells have been considered for adoptive immunotherapy and have yielded encouraging results in tumor therapy [9], [10]. The anti-tumor activity of CIK cells can be improved after co-culturing with dendritic cells (DCs) [11].

DCs are the most potent antigen-presenting cells in the body and can promote the generation of helper and cytotoxic T cells, and are also stimulators of effective T cells that can present tumor antigens to T lymphocytes and induce anti-tumor immune responses [12]–[15]. Thus, the combination of DCs and CIKs can lead to a remarkable increase in cytotoxic activity; and show more effective than single treatment [16], which has gained encouraging clinical prospects and has been widely used to treat solid and hematological system carcinomas [17], [18].

Meta-analysis based on data from pooled patient samples provides an avenue for evaluating the efficacy and side effects of chemotherapy combined with DC-CIK for advanced NSCLC patients. In this study, we used a meta-analysis to evaluate the efficacy and safety of the combination therapy on advanced NSCLC patients.

## Materials and Methods

### Literature Search Strategy

Electronic databases, including Cochrane Library, EMBASE, PubMed and Web of Science, were searched for studies that could be included in this meta-analysis from 2003 to 2014. Articles published in English and Chinese were enrolled. Search terms were “Dendritic Cells and Cytokine-Induced Killer Cells” or “DC-CIK immunotherapy”, “non-small-cell lung cancer” or “NSCLC”, and “Chemotherapy”. Our search based on PRISMA guidelines [19].

### Inclusion Criteria

Trials were eligible for inclusion in the present meta-analysis if they were randomized controlled trials (RCTs) of patients with advanced NSCLC. Patients in the control group received chemotherapy alone, whereas patients in the experimental group received chemotherapy combined with DC-CIK immunotherapy.

### Study Selection

The following selection criteria were used: (1) studies were written in English and non-English languages and limited to human trials (2) studies that performed and completed randomize controlled trials (RCTs).

### Quality Assessment

The quality of the included RCTs was assessed in accordance with the Cochrane Handbook [20] by recording seven items of bias risk: random sequence generation; allocation concealment; blinding of participants; blinding of outcome assessment; incomplete outcome data addressed; and free of selective reporting. Each of the seven items was scored as “low risk”, “unclear risk” or “high risk”.

### Data Extraction

Two independent reviewers (RXH, PP) scanned titles and available abstracts to identify potentially relevant articles. Disagreements were discussed with a third investigator (XL). The following data were collected: the first author's last name; the year of publication; the country where the study was performed; study design; number of years of follow-up period or study period; age range; number of subjects; and NSCLC stages.

### Curative Effects

Clinical responses were assessed in terms of the overall survival (OS) and progression free survival (PFS) to evaluate prognosis. Partial response (PR), overall response rate (ORR) and disease control rate (DCR) were considered to assess treatment efficacy. OS was defined as the time from the start of treatment to the time of death from any cause. PFS was defined as the length of time during and after treatment in which the patients lived with a disease that did not worsen. ORR was defined as the sum of partial and complete response rates, and the DCR was the sum of stable disease, partial response and complete response rates. These values were in accordance with the criteria provided by the World Health Organization.

### Safety Assessment

Adverse events (AEs) during the follow-up periods of all of the included studies were determined. AEs [21], [22] could be characterized as fatal, life threatening, required or prolonged existing hospitalization, or persistent or significant disability or indisposition and were graded in accordance with the criteria provided by the National Cancer Institute Common Toxicity [23].

### Statistical Analysis

Statistical analysis was performed using Review Manager Version 5.0 provided by the Cochrane Collaboration. *P*<0.05 was considered statistically significant. Heterogeneity [24] between trials was assessed to determine the most suitable model. Once heterogeneity was verified, a random-effect method was used; otherwise, a fixed-effect method was used. To evaluate whether or not the results of studies were homogenous, we performed Cochran's Q-test in which homogeneity was considered at *I* ^2^<50% or *P*>0.1. Risk ratios (RR) were the principal measures of effect and presented with a 95% confidence interval (CI). Sensitivity analysis was conducted and two trials (Wu et al. [25], Zhao et al. [26]) were excluded because DC immunotherapy was not applied in experimental group.

## Results

### Search Results

**Figure 1:**
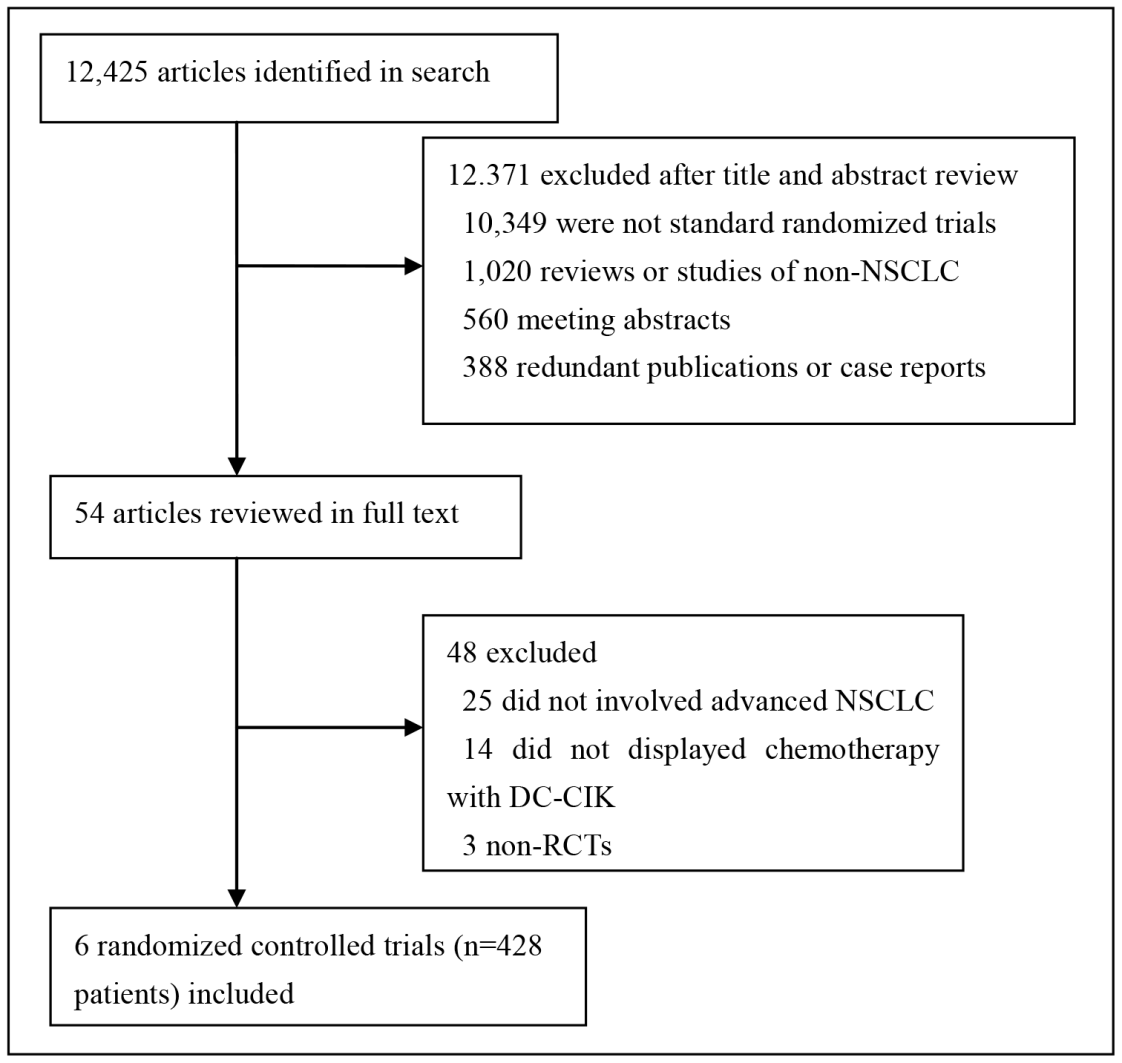
Flow diagram.

A total of 12,479 articles were identified during the initial search. By scanning titles and abstracts, redundant publications, reviews, meeting abstracts, and case reports were excluded. After referring to full texts, we removed 12,473 articles that did not satisfy the selection criteria: (1) not involved advanced NSCLC; (2) not displayed chemotherapy with DC-CIK immunotherapy; and (3) non-RCTs. As a result, 6 trials that included a total of 428 patients were eligible in the present analysis. The exclusion reasons were illustrated in **Figure 1**.

**Table 1** showed the characteristics of the six trials [25]–[30] included in the meta-analysis. All of the trials were conducted in mainland China. Among these trials, five provided the specific years of follow-up (two years to seven years). All of the six studies were randomized, and items were ranked as “low risk” based on the Cochrane Handbook.

**Table 1:** Clinical trials of the meta-analysis of advanced non-small-cell lung cancer (NSCLC).

Study	Patients (*N* = 428)	Gender (F/M)		Median Age (Range)		Follow up (Years)	Stages	Treatment Design	
		Exp	Con	Exp	Con			Exp	Con
Wu 2008[25]	59	7/23	5/24	61.0(38–74)	60.0(41–78)	3	IIIa/IIIb/IV	TP+CIK	TP
Zhao 2009 [26]	75	13/23	13/26	50.2(44–72)	51.3(42–68)	2	IIIa/IIIb/IV	NP+CIK	NP
Zhong 2011 [27]	28	8/6	7/7	No	No	7	IIIa/IIIb/IV	NP+DC/CIK	NP
Shi 2012 [28]	60	13/17	12/18	60.5(40–77)	58.5(40–76)	3	IIIa/IIIb/IV	NP+DC/CIK	NP
Yang 2012 [29]	122	12/49	12/49	63.0(29–80)	63.5(28–82)	2	IIIb/IV	NP+DC/CIK	NP
Li 2009 [30]	84	14/28	14/28	61.0(44–78)	60.5(40–80)	2	I/II/III	NP+DC/CIK	NP

**Note:** A total of 428 patients were included in the meta-analysis; among these patients, 213 were assigned to the experimental group (Exp) treated with DC-CIK/CIK plus Chemotherapy and 215 were assigned to the control group (Con) treated with chemotherapy alone.

**Abbreviations**: F, Female; M, Male, CIK, cytokine-induced killer biotherapy; DC, dendritic cells; NP, vinorelbine-platinum chemotherapy; TP, tocetaxel-cisplatin chemotherapy.

### Meta-Analysis of Prognosis Evaluation

The prognosis included two parts, namely, OS and PFS.

**Figure 2:**
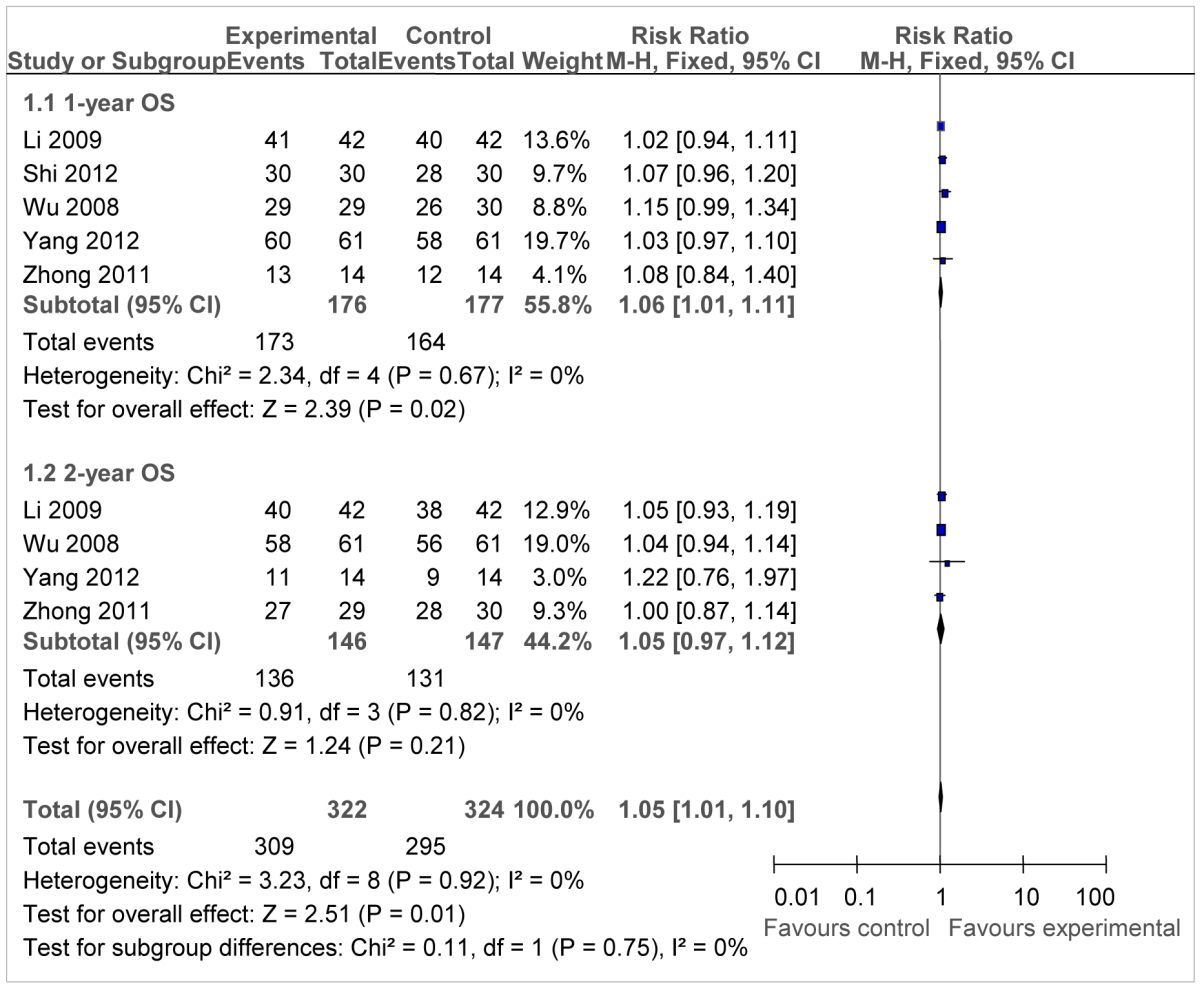
Forest plot of the comparison of overall survival (OS). *P* values are from P for the effect modification evaluation of heterogeneity within or across the groups of regimens. CI, confidence interval; RR, risk ratio; DC/CIK, DC-CIK immunotherapy; Chemo, chemotherapy; Con, control group; Exp, experimental group. A fixed-effect meta-analysis model (Mantel-Haenszel method) was used.

Among the six trials, five reported 1-year OS rate and four reported 2-year OS rate (**Figure 2**). Considering that slightly significant heterogeneity was detected, we selected the fixed-effect model. Chemotherapy combined with DC-CIK immunotherapy showed significant increase in 1-year OS compared with that of chemotherapy alone (RR = 1.06, 95%CI = 1.01–1.11, *P* = 0.02) according to the test for overall effect, however, the 2-year OS in the experiment group was not significantly different from those in control group (RR = 1.05, 95%CI = 0.97–1.12, *P* = 0.21).

**Figure 3:**
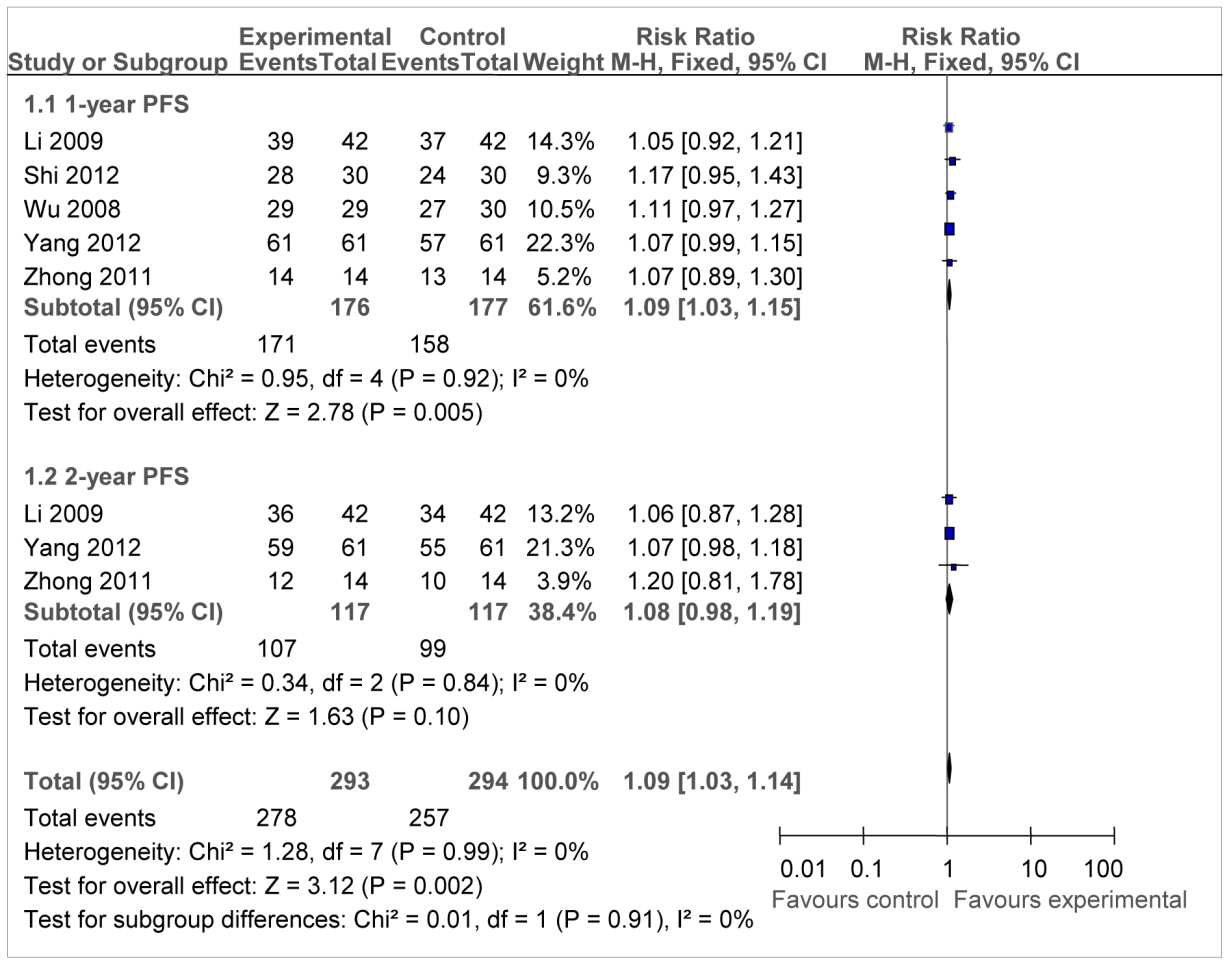
Forest plot of the comparison of progression free survival (PFS). *P* values are from P for the effect modification evaluation of heterogeneity within or across the groups of regimens. CI, confidence interval; RR, risk ratio; DC/CIK, DC-CIK immunotherapy; Chemo, chemotherapy; Con, control group; Exp, experimental group. A fixed-effect meta-analysis model (Mantel-Haenszel method) was used.

In terms of PFS, five studies presented relevant data of 1-year PFS and three reported 2-year PFS. In **Figure 3**, chemotherapy combined with immunotherapy significantly prolonged 1-year PFS (RR = 1.09, 95CI% = 1.03–1.15, P = 0.005) compared with chemotherapy alone. However, for 2-year PFS, the experimental group had no significant difference (RR = 1.08, 95CI% = 0.98–1.19, P = 0.10) compared with control group.

### Meta-Analysis of Efficacy Assessment

Efficacy was assessed in terms of DCR, ORR and PR.

**Figure 4:**
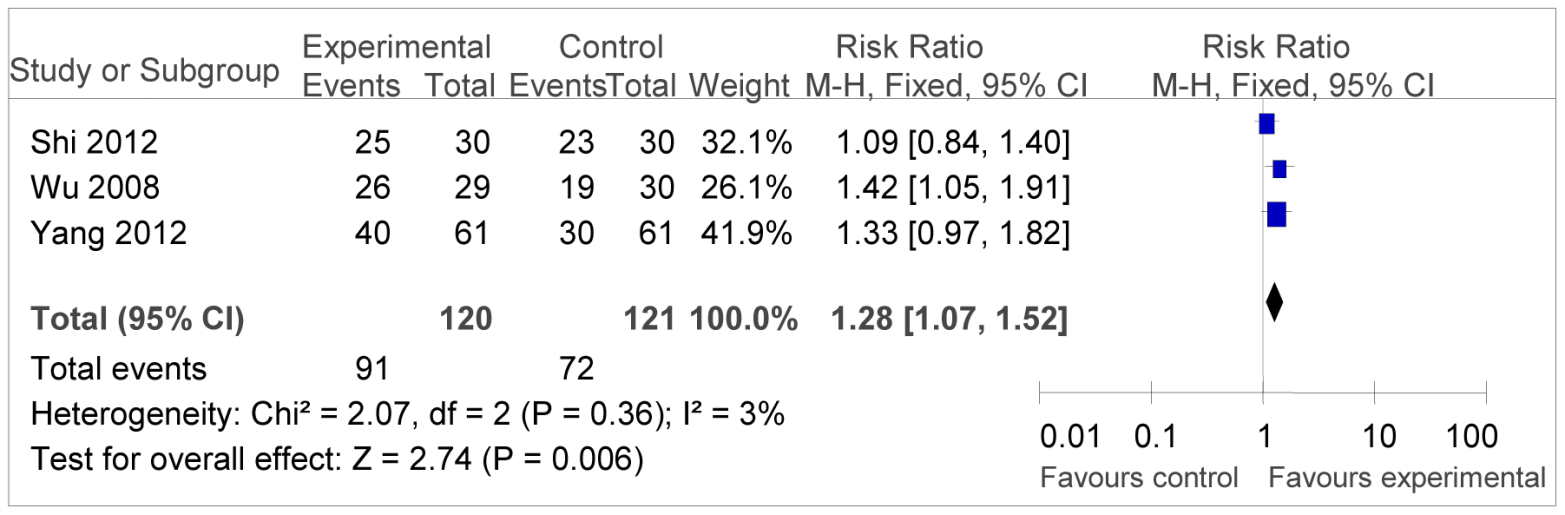
Forest plot of the comparison of disease control rate (DCR). *P* values are from P for the effect modification evaluation of heterogeneity within or across the groups of regimens. CI, confidence interval; RR, risk ratio; DC/CIK, DC-CIK immunotherapy; Chemo, chemotherapy; Con, control group; Exp, experimental group. A fixed-effect meta-analysis model (Mantel-Haenszel method) was used.

**Figure 5:**
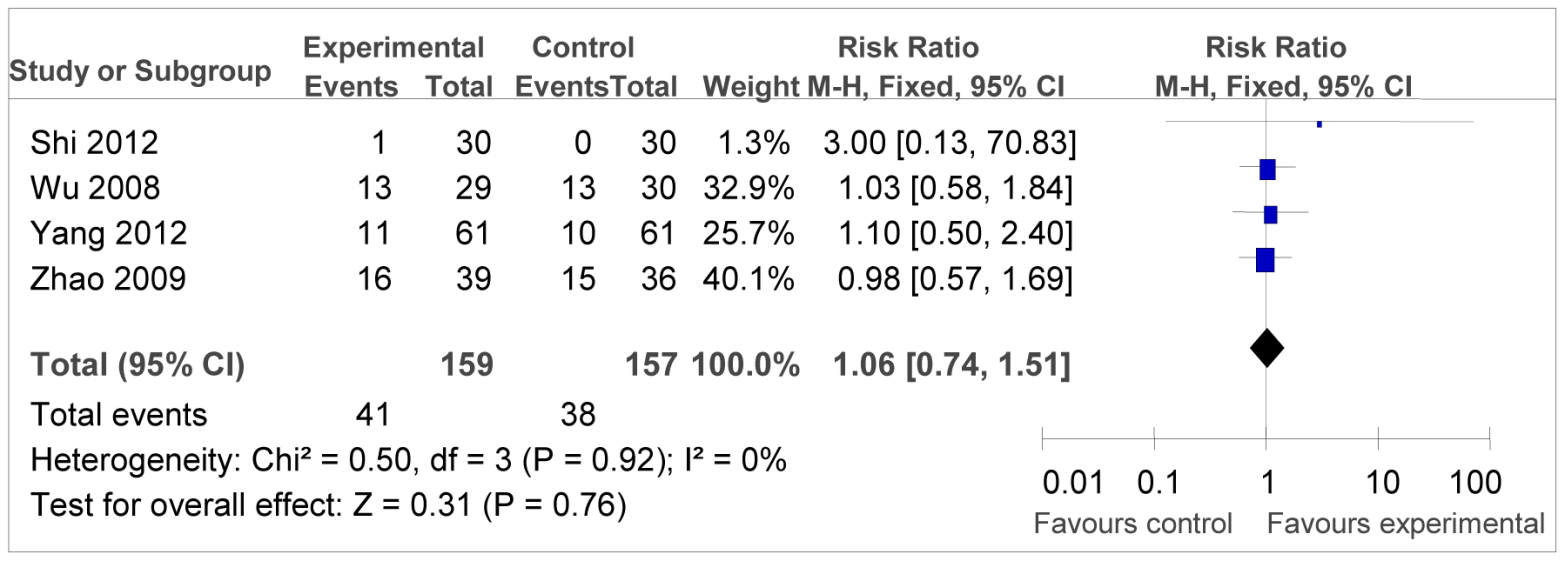
Forest plot of the comparison of overall response rate (ORR). *P* values are from P for the effect modification evaluation of heterogeneity within or across the groups of regimens. CI, confidence interval; RR, risk ratio; DC/CIK, DC-CIK immunotherapy; Chemo, chemotherapy; Con, control group; Exp, experimental group. A fixed-effect meta-analysis model (Mantel-Haenszel method) was used.

The analysis result of DCR was shown in **Figure 4**, revealing positive outcomes for the combination therapy (RR = 1.20, 95% CI = 1.07–1.52, *P* = 0.006). But the RR of ORR was 1.06 (95% CI = 0.74–1.51, *P* = 0.76), which showed in **Figure 5**, did not infer significantly difference between two groups.

**Figure 6:**
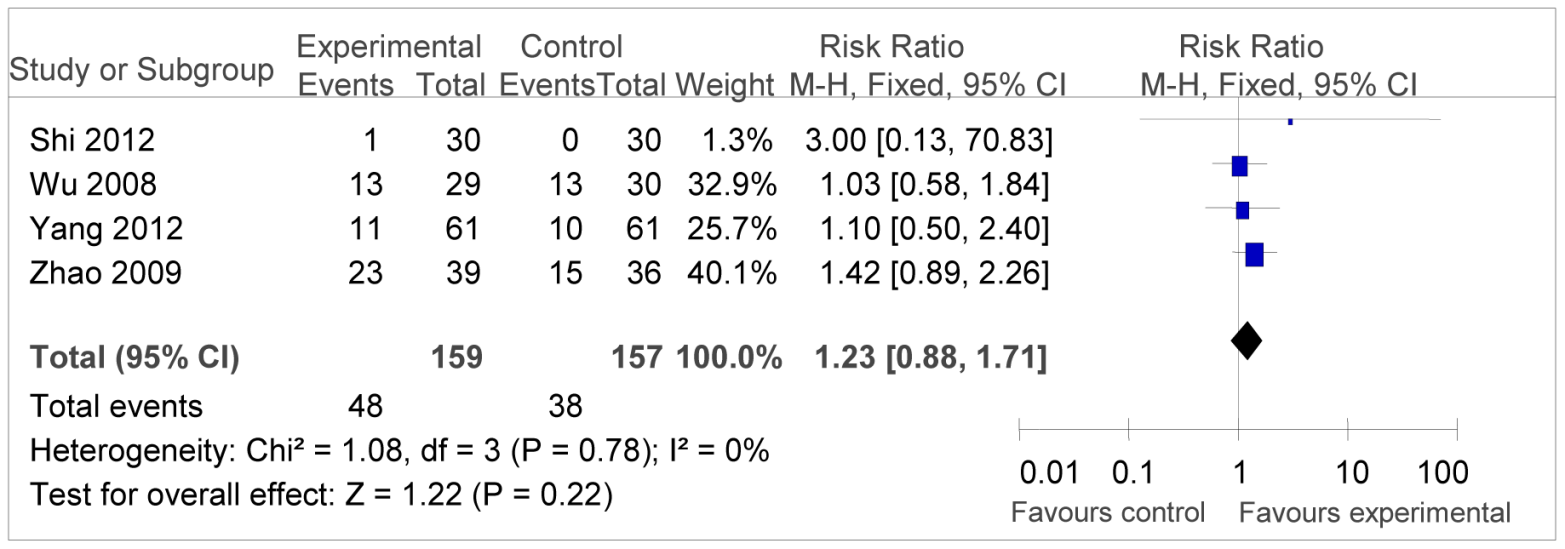
Forest plot of the comparison of partial response rate (PR). *P* values are from P for the effect modification evaluation of heterogeneity within or across the groups of regimens. CI, confidence interval; RR, risk ratio; DC/CIK, DC-CIK immunotherapy; Chemo, chemotherapy; Con, control group; Exp, experimental group. A fixed-effect meta-analysis model (Mantel-Haenszel method) was used.

Fix-effect models were chosen to analyze the PR rate because low heterogeneity was obtained. In **Figure 6**, RR was 1.23 (95% CI = 0.88–1.71, *P* = 0.22), suggesting no statistically significant improvement between two groups.

### Sensitivity Analysis

Considering that not all of the **efficacy** parameters were presented in all of the reviewed studies, we performed sensitivity analyses separately on each parameter in accordance with the alternative exclusion criteria of trials, such as the studies by Wu et al [25] and Zhao et al [26], which did not apply the DC method. The results of this analysis were similar to those obtained from the overall analysis of the pooled trials.

### Assessment of AEs or Toxicity for advanced NSCLC

The current clinical trials with advanced NSCLC indicated considerable AEs or toxicity. The details of treatment-related AEs or toxicity were summarized in **Table 2**.

**Table 2:** Adverse events in advanced non-small-cell lung cancer (NSCLC).

Adverse Events	Wu et al.		Li et al.		Yang et al.		Zhao et al.		Shi et al.		Zhong et al.	
	*N* = 59		*N* = 84		*N* = 122		*N* = 75		*N* = 60		*N* = 28	
Groups	Exp	Con	Exp	Con	Exp	Con	Exp	Con	Exp	Con	Exp	Con
Leukopenia	-	-	-	-	-	-	48.7%	83.3%	-	-	71.4%	92.8%
Nausea	-	-	-	-	-	nc	51.2%	86.1%	-	-	64.2%	92.8%
Anemia	-	-	nc	-	nc	nc	17.8%	44.5%	-	-	28.5%	42.8%
Insomnia	-	-	-	-	-	-	7.7%	30.6%	-	-	-	-
Temporary Fever	nc	-	nc	-	nc	nc	-	-	13.3%	nc	71.4%	21.4%
Headache	nc	-	-	-	-	nc	-	-	-	-	-	-
Fatigue	-	-	-	-	-	-	-	-	10.0%	nc	7.1%	57.1%
Thrombocytopenia	-	-	-	-	-	-	20.5%	38.9%	-	-	-	-
Chest distress	-	-	-	-	-	-	-	-	3.3%	nc	-	-

**Abbreviations:** Exp: experimental group; Con: control group; Nc: not clear, that is, simply mentioned in the article but did not provide an exact number; -, no description.

In **Table 2**, all of the six trials reported adverse effects. However, three of these trials [25], [27], [30] did not provide the exact numbers of AEs. In both groups, leukopenia, nausea, anemia, insomnia, temporary fever, headache, fatigue, thrombocytopenia, and chest distress were observed. Among them, temporary fever, anemia, leukopenia and nausea were the four main AEs.

The results indicated that chemotherapy combined with DC-CIK therapy could obviously alleviate leukopenia, nausea, anemia, insomnia, fatigue, and thrombocytopenia compared with chemotherapy alone. For temporary fever, the experimental group was a little more than the control group and could be relieved naturally in 24 hours without any medical treatment.

For chest distress, the effectiveness of chemotherapy combined with DC-CIK remained unclear because chemotherapy alone was not clearly described.

## Discussion

The 6 trials included in this meta-analysis adopted chemotherapy combined DC-CIK therapy for patients with advanced NSCLC. Hence, the number of published RCTs would affect the results of this study and the quality of the reported data influenced the power of our meta-analysis, and greater statistical reliability would be achieved if additional and more comprehensive trials including all of the efficacy parameters were enrolled. Nevertheless, sensitivity analysis supported the conclusions drawn from the overall unstratified analyses.

Other factors, such as individual difference of patients, different lengths of follow-up may confer limitations on this meta-analysis. In overall studies, no significant publication bias existed, in addition, as many RCTs as possible were included to improve the statistical reliability. Our literature search strategy guaranteed that there was less possibility of important published trials being overlooked. According to our meta-analysis, all patients with advanced NSCLC met quality-control specifications and protocol eligibility. Finally, risk ratios demonstrated that no statistical inconsistency existed between results from each of the original studies and those of overall efficacy suggested that the results were valid.

For clinical therapy, effectiveness and safety are the key factors [31]. At present, DC-CIK technology is widely used in clinic due to its higher security. Up to date, a large body of clinical evidence indicated that there was neither serious AEs nor death caused by DC-CIK therapy. The main side effects are temporary fever (usually below 39°C) and cold symptoms [32].

The present meta-analysis indicated that chemotherapy combined with DC-CIK had potential advantages in NSCLC treatment: firstly, its efficiency was observed in clinic. An outstanding characteristic was significant increase in 1-year OS (*P* = 0.02) and 1-year PFS (*P* = 0.005). Besides, DCR in the combined therapy was also improved significantly (*P* = 0.006), and patients obtained better quality of life, such as relieving pain, fatigue and insomnia; secondly, the AEs of chemotherapy combined with DC-CIK were alleviated obviously compared with that of the chemotherapy alone, including leucopenia, nausea, anemia, insomnia, fatigue, and thrombocytopenia. Undoubtedly, these were the greatest benefits for patients.

However, the efficacy of chemotherapy combined with DC-CIK has been in argument, especially in long-term effectiveness. The analysis of 2-year OS (*P* = 0.21) and 2-year PFS (*P* = 0.10) showed no statistical significance between the two groups. For ORR (*P* = 0.76) and PR (*P* = 0.22), there appeared no statistical differences, too. These results suggested that the current DC-CIK immunotherapy is modest in efficacy. This may be related to large tumor burden in the advanced NSCLC as well as the shortages of the methods for generation of DC and CIK. It is possible that (a) current methods for generation of DCs are unable to generate sufficient number of immunogenic DCs; (b) these DCs are unable to efficiently process and present endogenous tumor antigens, and (c) CIKs are short on life if endogenous and exogenous DC could not provide sufficient help for their survival.

Taken together, although chemotherapy combined with DC-CIK is a recommendable method and applies successfully in clinic for patients with NSCLC [33], [34], our meta analysis indicates that this type of therapy currently is modest for NSCLC. Especially, the quality of DC-CIK needs to be rigorously improved to enhance therapeutic efficacy and prolong the survival period of patients.

## Supporting Information

Checklist S1 **PRISMA Checklist.**
(DOC)
